# Dexamethasone Alters Tracheal Aspirate T-Cell Cytokine Production in Ventilated Preterm Infants

**DOI:** 10.3390/children8100879

**Published:** 2021-10-02

**Authors:** Siamak M. Yazdi, Ekta U. Patel, Colby D. Richardson, K. Thomas Hardy, John E. Baatz, Jennifer K. Mulligan, Rita M. Ryan

**Affiliations:** 1 Department of Pediatrics-Neonatology, Medical University of South Carolina, Charleston, SC 29425, USA; eupatel@cmh.edu (E.U.P.); hardykr@musc.edu (K.T.H.); baatzje@musc.edu (J.E.B.); Rita.Ryan@uhhospitals.org (R.M.R.); 2 Department of Pediatrics-Neonatology, University of Alabama at Birmingham, Birmingham, AL 35294, USA; 3 Department of Pediatrics-Neonatology, University of Missouri-Kansas City School of Medicine, Kansas City, MO 64108, USA; 4 Department of Pediatrics-Neonatology, University of Rochester Medical Center, Rochester, NY 14642, USA; Colby_Richardson@URMC.Rochester.edu; 5 Department of Otolaryngology, Medical University of South Carolina, Charleston, SC 29425, USA; Jennifer.Mulligan@medicine.ufl.edu; 6 Division of Pulmonary, Critical Care and Sleep Medicine, University of Florida Gainesville, Gainesville, FL 32611, USA; 7 Department of Pediatrics-Neonatology, Case Western Reserve University, UH Rainbow Babies and Children’s Hospital, Cleveland, OH 44106, USA

**Keywords:** immunology, inflammation, bronchopulmonary dysplasia, lung, Neonatology, Pulmonology

## Abstract

Postnatal corticosteroids improve respiratory status and facilitate respiratory support weaning in preterm infants with bronchopulmonary dysplasia (BPD). Older literature describes characteristic cytokine profiles in tracheal aspirates (TA) of BPD patients which are altered with corticosteroids. Corticosteroids also influence peripheral blood T-cell presence. However, little is known regarding TA T-cell phenotype and cytokine production before or after exogenous corticosteroids. We hypothesized that postnatal dexamethasone alters the TA T-cell cytokine profiles of preterm infants. TA samples were collected from 14 infants born from 23 0/7 to 28 6/7 weeks who were mechanically ventilated for at least 14 days. Samples were collected up to 72 h before a ten-day dexamethasone course and again 1 to 3 calendar days after dexamethasone initiation. The primary outcome was change in T cell populations present in TA and their intracellular cytokine profile after dexamethasone treatment, ascertained via flow cytometry. Following dexamethasone treatment, there were significant decreases in respiratory severity score (RSS), percent CD4+IL-6+ cells, CD8+IL-6+ cells, CXCR3+IL-6+ cells, and CXCR3+IL-2+ cells and total intracellular IFN-γ in TA. RSS significantly correlated with TA percent CD4+IL-6+ cells. To our knowledge, this is the first study demonstrating that dexamethasone reduced T-cell IL-6 and this reduction was associated with improved RSS in pre-term infants with evolving BPD.

## 1. Introduction

Postnatal corticosteroids are used in preterm infants with evolving or established bronchopulmonary dysplasia (BPD) with the aim of reducing inflammation in the lung to improve respiratory status and facilitate weaning of respiratory support (for review see Htun et al. [1]). Previous studies demonstrate that corticosteroid treatment leads to decreased duration of ventilation, decreased oxygen requirement, and improved pulmonary function [1,2,3,4,5]. When compared to hydrocortisone and methylprednisolone, dexamethasone therapy has been shown to have a slightly greater benefit to short-term respiratory outcomes by day 7 as assessed by reduction in respiratory severity score (RSS, defined as the mean airway pressure multiplied by the fractional inspired content of oxygen) [6]. Corticosteroids have numerous anti-inflammatory and immunomodulating effects, which may explain their benefits in ventilator-dependent preterm infants.

Although the complex pathophysiology of BPD cannot be fully explained by inflammatory factors alone, there is evidence that early elevation of proinflammatory cytokines in the interleukin (IL) family (including IL-1β, 6, 8, 16) may be predictive of infants who later develop BPD [7,8]. It has been previously shown that infants with BPD have increased concentrations of pro-inflammatory cytokines such as IL-6 in tracheal aspirates (TA), which are subsequently reduced after corticosteroid treatment [9,10]. Furthermore, elevated cord blood IL-6 is an early predictor for the later development of BPD [11]. Other studies have also demonstrated the utility of TA analysis in investigating BPD, such as the finding that infants with a higher concentration of the transcription factor nuclear factor kappa B (NF-κB) in TA during the initial 3 days of life are more likely to develop BPD [12], and that higher levels of SPARC (Secreted Protein, Acidic, Rich in Cysteine) in the first week conferred a higher risk of BPD development or death [13].

Systemic dexamethasone therapy for BPD patients has also been shown to alter certain peripheral blood lymphocyte populations, notably a decrease in CD4+T-cells [2]. CD4+T-cells have diverse roles and subsets, active in innate and adaptive immune function and regulation [14]. Interestingly, peripheral CD4+T-cells have also been shown to be significantly lower in premature infants who eventually develop BPD when measured during the first two weeks of life, whereas other peripheral blood lymphocyte populations, such as CD8+ T-cells, lack such differences [15]. CXCR3, a chemokine receptor highly expressed on type 1 helper (Th1) T-cells, represents another area of interest in T-cell mediated inflammation. CXCR3 expression regulates trafficking of Th1 cells to injured tissue to amplify the inflammatory response [16]. Furthermore, a large longitudinal cohort study demonstrated that T-cell phenotype at birth was influenced by gestational age, with CD4+ T-cells transitioning from CD31– TNF-α+ mid-gestation toward a CD31+IL-8+ phenotype closer to full term gestation. Preterm infants low in CD31+IL8+CD4+T cells at discharge were found to be at higher risk for post-discharge respiratory complications, emphasizing the powerful role of T-cell function in respiratory morbidity and mortality of preterm infants [17].

There is limited understanding of the significance of T-cell expression profiles and cytokines in the lungs of ventilated preterm infants. We hypothesized that the administration of postnatal dexamethasone to ventilated preterm infants reduces the pro-inflammatory nature of T-cells as measured by intracellular cytokine production. We used a panel of T-cell markers and to specifically examine expression on T-cells of common pro-inflammatory cytokines, since these studies were exploratory in a small cohort of patients. T-cells were studied because CD3+ T-cells were shown in previously unpublished but nationally presented data to be more prevalent in the lungs of deceased infants with BPD compared with similar corrected gestational age infants who died with no lung disease [18]. CXCR3 was studied based on its known association with adult idiopathic fibrosis [19]. IL-6 was included because higher TA IL-6 on day 3 of life is associated with later BPD [20]. If our hypothesis is confirmed, better description of cytokine expression and receptor changes may elucidate the mechanism of dexamethasone positively influencing respiratory outcomes in these infants. Characterization and clinical correlation of these factors may enable improved decisions regarding the timing, initiation, and duration of corticosteroids in ventilator-dependent preterm infants, or perhaps inform more specific treatments, sparing the use of steroids with their broad range of effects and side effects.

## 2. Materials and Methods

### 2.1. Ethics

This study was performed with the approval of the Medical University of South Carolina Institutional Review Board (IRB Protocol 00018389, approved 13 August 2012). All subjects’ parents provided written informed consent.

### 2.2. Patient Characteristics

This pilot study utilized a prospective observational cohort with convenience sampling. Infants were selected for inclusion if they were born between 23 0/7 weeks and 28 6/7 weeks, and mechanically ventilated for at least 14 days prior to initiation of dexamethasone. Infants who received any prior corticosteroids or had any life-threatening congenital anomalies were excluded. Samples were obtained from 1 October 2012 to 30 April 2019 for infants who met the inclusion criteria. Prophylactic surfactant was given routinely for all inborn infants <28 weeks gestational age prior to 31 January 2019 and to those <26 weeks gestational age after 1 February 2019. Only one patient was enrolled following this change, and as this infant was born at 25 weeks and 6 days, the change in protocol would not have changed their care. Rescue surfactant was given to any infant diagnosed with respiratory distress syndrome requiring continuous positive airway pressure (CPAP) and at least 30% FiO2. Basic demographic information was obtained (Table 1). Infants who were included all ultimately met criteria for BPD per the NIH 2001 National Institute of Child Health and Development workshop definition of requiring supplemental oxygen use for greater than 28 days and assessment at 36 weeks postmenstrual age [21].

### 2.3. Dexamethasone Treatment and Tracheal Aspirate Sample Collection

Infants were selected for dexamethasone therapy based on the discretion of the clinical team in our neonatal intensive care unit (NICU), independent of this study. A 10-day tapering course of dexamethasone published by Doyle et al. was used [22]. TA were obtained during routine, clinically-indicated suctioning by the bedside nurse or respiratory therapist, with a 1 mL saline lavage. Infants had TA obtained up to 72 h prior to initiation of the 10-day dexamethasone course and then a subsequent TA collection 1 to 3 calendar days after dexamethasone was initiated. A total of 14 infants were included for the study based on usable sample availability. All TA obtained were placed at 4 °C for up to 2 h until they were transported to the laboratory for processing. During the processing, cells and lavage fluid were separated by centrifugation at 500 g for 10 min. Cells were cryopreserved in 90% FBS/10% DMSO freezing media and stored in liquid nitrogen.

### 2.4. Respiratory Severity Score

Clinical Respiratory Severity Score (RSS) was calculated on day 0 (prior to dexamethasone initiation) and day 3 (72 h after dexamethasone initiation). RSS was defined as the mean airway pressure multiplied by the fractional inspired content of oxygen. Chart review was utilized to identify the mean airway pressure and fractional inspired content of oxygen at the time of dexamethasone initiation and 72 h thereafter. 

### 2.5. Immunostaining and Flow Cytometric Analysis 

Immune cell phenotyping was conducted by intracellular immunostaining with flow cytometric analysis using previously described methods [23,24,25,26,27]. The primary outcome was change in T-cell cytokine expression after dexamethasone treatment, specifically CD4, CD8, and CXCR3 T-cells and their respective expression of interferon-γ (IFN-γ), IL-2, and IL-6. The TA cells were thawed, washed in fluorescence-activated cell sorting (FACS) Buffer with FACS Block (FACS Buffer plus bovine serum albumin) supplemented with 10 µL/mL Human FC Block (eBioscience, San Diego, CA, USA). All antibodies (supplemental Table 1) were purchased from BD Biosciences (Franklin Lakes, NJ, USA). Extracellular markers included CD4 (557871), CD8 (557746) and CXCR3 (551128). Live cells were identified by Zombie Live/Dead stain (eBioscience). Prior to intracellular staining, cells were permeabilized using transcription factor staining buffer (eBioscience, 00-5521). Analysis of intracellular cytokines included Interferon-gamma (IFN-γ) (554702), Interleukin (IL)-2 (559334), and IL-6 (554544). Samples were assayed immediately using a Guava 8 HT flow cytometer (Luminex, Austin, TX, USA) and analyzed with FCS Express 5.0 (DeNovo Software, Tibco, Palo Alto, CA, USA). Dead cells were excluded from the final data analysis. The percent of live cells ranged from 38–83% viable with a mean percent viable of 56.9%. The percent of viable cells did not change with dexamethasone treatment, nor was it associated with any of measured outcomes. Marker gates were set using matched isotype controls with isotype subtraction was performed on all samples. 

### 2.6. Statistical Analysis

Standard statistical analyses for outcomes were conducted using GraphPad Prism 7 (GraphPad Software, La Jolla, CA, USA). The pretreatment sample subset served as self-controls and was compared to values obtained up to 72 h following treatment. A D’Agostino and Pearson omnibus test was used to determine if data sets were normally distributed. Since some of the data sets were not normally distributed (presented as median (range) rather than mean (standard deviation (SD)), for all data sets, a two-tailed Wilcoxon matched-pairs signed rank test was applied. Values were deemed statistically significant when *p* < 0.05. 

## 3. Results

There was a wide range of birth weights and weights at time of treatment, as well as an array of gestational ages present. Twenty-eight TA samples from 14 patients (pre- and post-dexamethasone) were included in this study after applying inclusion and exclusion criteria. These 14 infants were born at a median of 25 6/7 weeks postmenstrual age (range of 23 1/7–27 3/7 weeks) and mean of 772 g (range of 540–1250 g) but were a median of 29 5/7 weeks postmenstrual age (range 24 6/7–37 6/7 weeks) with a mean current weight of 1157 g (range of 595–2310 g) at the time of dexamethasone treatment (Table 1). The distribution of sex and race reflects our NICU preterm population with BPD; the patients included are predominately male and black (Table 1). 

RSS was calculated on day 0 prior to dexamethasone initiation and on day 3 of the dexamethasone course. Mean (SD) pre-treatment RSS was 7.21 (3.94); mean post-treatment RSS was 5.28 (3.47), *p* = 0.0005 by two-tailed, paired Wilcoxon matched-pairs signed rank test (Table 1). Mean RSS reduction at day 3 was 1.94 (standard deviation of 1.74). 

**Figure 1:**
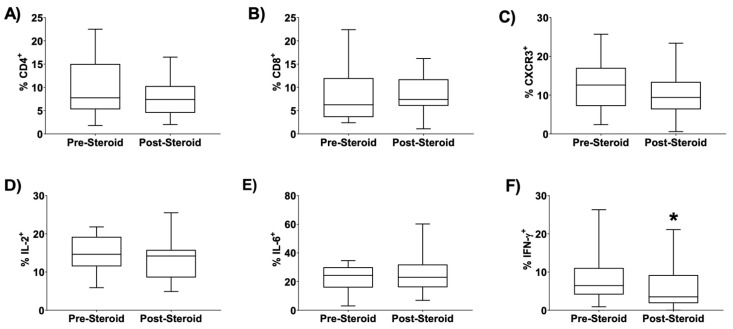
Effect of Dexamethasone on TA Cytokine Expression and T-Cell Composition (**A**–**F**). Dexamethasone significantly reduced tracheal aspirate (TA) IFNγ^+^ cells. (Figure 1F, * *p* = 0.01), but not IL-2^+^ or IL-6^+^ cells), and did not affect T-cell composition when assessed by flow cytometry. Statistical analysis performed by two-tailed, paired Wilcoxon matched-pairs signed rank test, *n* = 14. Lines in the box and whisker plots represent the median, box extends from the 25th to 75th percentile, and the bars represent range.

Flow cytometry was utilized on the pre- and post-dexamethasone treatment samples to quantify the relative presence of T-cell subtypes, specifically T-cells expressing CD4, CD8, or CXCR3, as well as the expression of the Th1 cytokines, IL-2, IL-6, and IFN-γ. TA T-cell subtype composition remained unchanged post-dexamethasone course initiation as indicated by flow cytometry of T-cells expressing CD4, CD8, or CXCR3 (Figure 1A–C). Similarly, total expression of IL-2 and IL-6 remained unchanged post dexamethasone course initiation (Figure 1D,E). However, post-dexamethasone initiation, there was a significant reduction in the percent total cells expressing IFN γ (*p*= 0.01 by two-tailed, Wilcoxon matched signed rank test, *n* = 14) (Figure 1F). 

**Figure 2:**
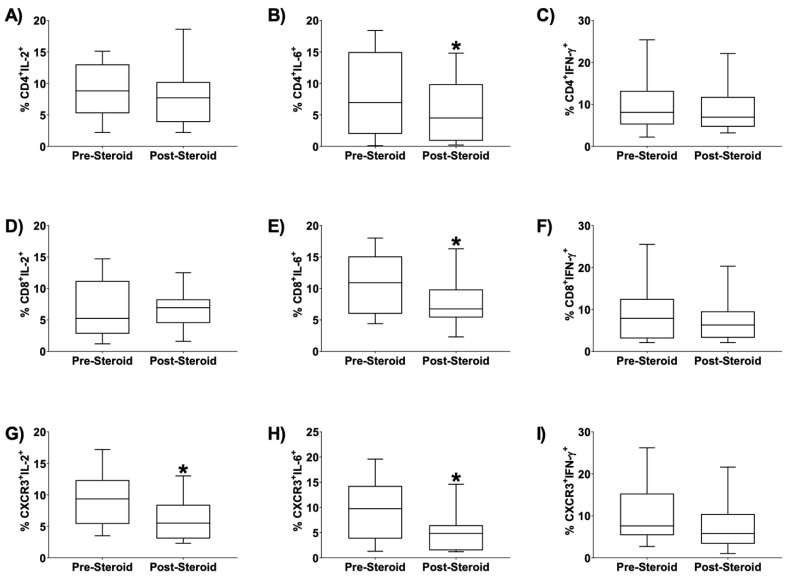
Effect of Dexamethasone on TA T-Cell Subpopulations Expressing IL-2, IL-6, and IFNγ (**A**–**I**). Several subpopulations of tracheal aspirate (TA) T-cells were significantly altered after dexamethasone initiation. Flow cytometry demonstrated a significant reduction in CD4^+^IL-6^+^(panel B, * *p* = 0.01), CD8^+^IL-6 ^+^(E, * *p* = 0.01), CXCR3^+^IL-2^+^ (G, * *p* = 0.04), and CXCR3^+^IL-6^+^ (H, * *p* = 0.006) live cells in TA of mechanically ventilated preterm infants following dexamethasone initiation. Infants had TA obtained up to 72 h prior to initiation of the 10-day dexamethasone course and then a subsequent TA collection 1 to 3 calendar days after dexamethasone was initiated. *n* = 14. Statistical comparisons generating reported *p* values were by Wilcoxon signed-rank test.

We also evaluated the effect of dexamethasone initiation on the expression of cytokines for each T cell subtype being studied. Data revealed a significant reduction in the percent of CD4+ IL-6+ cells after dexamethasone initiation (Figure 2B, *p* = 0.01); a significant reduction in the percent of CD8+ IL-6+ cells after dexamethasone initiation (Figure 2E, *p* = 0.01); a significant reduction in the percent of CXCR3+ IL-2+ cells after dexamethasone initiation (Figure 2G, *p* = 0.04); and a significant reduction in the percent of CXCR3+ IL-6+ cells after dexamethasone initiation (Figure 2H, *p* = 0.006). No significant change was found in CD4+ IL-2+, CD4+ IFNγ+, CD8+ IL-2+, CD8+ IFNγ+, or CXCR3+ IFNγ+ live cells after dexamethasone initiation (Figure 2). Statistical comparisons generating reported *p* values were by Wilcoxon signed-rank test, *n* = 14.

**Figure 3:**
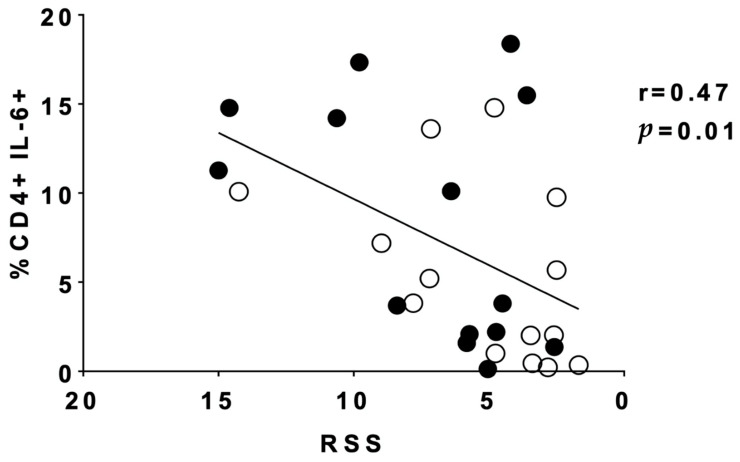
Tracheal aspirate (TA) cell phenotype of CD4+IL-6+ cells was significantly correlated with respiratory severity score (RSS, MAP × FiO2). As RSS decreased, the percent of CD4+IL-6 cells decreased, suggesting alterations in inflammation represented by CD4+IL-6+ expression are important for clinical respiratory status. Infants had TA obtained up to 72 h prior to initiation of the 10-day dexamethasone course and then a subsequent TA collection 1 to 3 calendar days after dexamethasone was initiated. *n* = 14. RSS was calculated on day 0 prior to dexamethasone initiation and 72 h later. Correlation was measured by Pearson’s correlation. Black circles indicate samples pre-dexamethasone. Open circles indicate samples post-dexamethasone.

Additionally, we examined whether any clinical correlations existed for the significant findings in changes of T-cell cytokine expression. When plotting RSS vs. the percentage of CD4+IL-6+ cells obtained for every patient both pre- and post- steroid initiation, we noted a statistically significant positive correlation (Figure 3, r = 0.47, *p* = 0.01). This suggests that a higher RSS is correlated with increased relative presence of CD4+/IL-6+ cells. These data suggest that dexamethasone treatment influences T-cell IL-6 secretion, which is associated with a reduction in RSS.

## 4. Discussion

In this pilot study, we aimed to explore whether treatment with dexamethasone leads to a change in T-cell populations or cytokine expression in TA of mechanically ventilated premature infants. The resulting data support our hypothesis that dexamethasone treatment does indeed alter TA T-cell cytokine expression. Corticosteroid therapy for BPD has been investigated in numerous studies, with evidence that it can facilitate the weaning of respiratory support and improve respiratory status [1,2,3,4,5]. Corticosteroids act as powerful anti-inflammatory agents, but there remains a need to fully elucidate the mechanisms by which corticosteroids alter pulmonary physiology and inflammation.

Although previous research suggests alterations in cytokine profile of TA with corticosteroid treatment, such as a reduction in the proinflammatory cytokine IL-6 [9], our study is unique in identifying T-cells in preterm infant TA and establishing the TA T-cell changes that result from dexamethasone therapy. More studies will have to be done to fully understand this effect of dexamethasone, with a focus on the CXCR3, CD4+, and IL-6 pathways, as well as delineation of whether the decrease in IFN-γ expression was due to T-cells, or rather related to other cell types that can produce IFN-γ such as NK cells. Furthermore, recent research with flow cytometry and TA demonstrates monocyte-specific cytokine pathways that change over time in infants at risk for BPD, providing another potential area for study to investigate how corticosteroid treatment influences monocytes and their function in BPD development [28].

Our patients had a drop in RSS at day 3 that was similar to that previously reported [6]. We identified a correlation between dexamethasone treatment and percent of CD4+ IL-6+ cells and also a correlation between RSS and percent CD4+IL6+ cells. This is an important clinical relationship, linking worse respiratory status with the particular T-cell cytokine subpopulations of higher CD4+IL-6+ cell presence, which presumably is more pro-inflammatory due to the expression of IL-6. It is not clear whether this means that infants with sicker lungs have more CD4+/IL-6+ cells contributing to their worse respiratory status, or if the presence of fewer CD4+/IL-6+ cells causes the respiratory status to improve. However, these findings support the hypothesis that the dexamethasone-induced decrease in pro-inflammatory T-cells, specifically CD4+IL-6+ cells, correlates with clinical respiratory improvement, and suggests a mechanism for the positive effects of dexamethasone in this context. Determining T-cell cytokine profiles that demonstrate a favorable response to corticosteroid therapy could enable identification of infants who would benefit most from a corticosteroid course. It is unsurprising that CD4+ T-cells expressing IL-6 are reduced by dexamethasone, a powerful anti-inflammatory drug. However, not all pro-inflammatory cytokine profiles we evaluated changed after dexamethasone. Further analysis about CD4+IL-6+ cells is needed to understand this unique response.

The lack of change in CD4+ cells in TA after dexamethasone is surprising, as it contrasts with previously published findings regarding peripheral blood lymphocyte populations in which dexamethasone diminished the presence of CD4+ cells, suggesting different physiology may govern the effects of dexamethasone in the lungs [2]. Alternatively, examining TA at 1 to 3 days post dexamethasone initiation may not have allowed for sufficient time to detect changes in immune cell infiltrate. Furthermore, CD8+ cells in the TA did not change after dexamethasone, a consistency which aligns with literature demonstrating similar CD8+ cell presence in the peripheral blood of premature infants during the first two weeks of life regardless of whether they later develop BPD [15].

The T-cell cytokine profiles that we determined to exhibit attenuation with dexamethasone administration may represent therapeutic targets for BPD therapy, an appealing proposition given the risks of corticosteroid therapy such as possible adverse neurodevelopmental outcomes [5], possible interference with standard immunizations, or standard drug side effects. The reduction of the pro-inflammatory population of CXCR3+ T-cells (with either IL-2 or IL-6 co-expression) suggests that migration of pro-inflammatory agents is influenced by this potent chemokine receptor. For example, interferon gamma-induced protein 10 (IP-10), a CXCR3 ligand, has been found in higher amounts in the lungs and airways of a baboon model of BPD when compared to control animals [29]. The bronchoalveolar lavage samples of adults with idiopathic pulmonary fibrosis exhibit comparatively less CXCR3+ cells than healthy controls [19], supporting a critical role for CXCR3 in chronic lung diseases. Antagonism of CXCR3 may provide an avenue of blunting pulmonary inflammation in BPD that avoids the potential risks of corticosteroids [5]. However, development of CXCR3 antagonists has proved challenging, without any current FDA-approved agents, though similar chemokine receptors antagonists such as plerixafor, a CXCR4 antagonist, have found clinical applications [30]. One CXCR3 antagonist, AMG 487, has been studied in psoriasis and graft vs. host disease [31,32]. Further investigation should focus on whether there is a potential role for CXCR3 blockade in illnesses involving pulmonary inflammation such as BPD.

The primary limitation of our study is the small number of samples (28) and subjects (14). Additional limitations of the study include the wide range of postmenstrual ages of the study subjects at the time of sampling and the potential risk of selection bias given the convenience sampling. Interpretation of our data without a true control group (e.g., placebo-treated) provides another limitation. However, our study does have the advantage of each subject being his or her own control, which decreases biological variability, suggesting the effects found are more likely due to the only change over 1 to 3 days of dexamethasone treatment. We did not note any other intervening confounders such as acute infection (e.g., pneumonia) in any of these subjects during the steroid course that could contribute to a change in T-cell populations. A larger sample size with more frequent sampling and perhaps a later time point collection would provide more granularity to the data, and assessing the concentrations of cytokines such as IL-2 and IL-6 in the TA would enable better understanding of the progression of inflammation. Sample collection timing proved challenging, as ideally the pre- and post-dexamethasone samples would be from precisely the same timepoint across all patients rather than the broader timespans that we used due to our convenience sampling.

Another limitation involving our significant findings related to IL-6 is that we only focused on T-cell IL-6, while other cell types including monocytes, macrophages, fibroblasts, epithelial cells and endothelial cells can also produce this cytokine. The total percent of live cells expressing IL-6 did not change, which supports the notion that our significant findings involved T-cells and their response to dexamethasone. Additionally, some epithelial, NK, and dendritic cells can express CXCR3, for which this study was not designed to control [33].

In summary, our study demonstrates the feasibility of measuring T-cell subpopulations from tracheal aspirates from mechanically ventilated preterm infants. We demonstrated that dexamethasone reduced respiratory support as expected, while uncovering TA T-cell changes that are novel downstream anti-inflammatory effects of corticosteroid use in BPD. Using our data to focus future studies on the T-cell populations that express IL-6 or CXCR3 might be helpful in identifying future specific targets to decrease lung inflammation in infants with evolving BPD.

## Figures and Tables

**Table 1:** Demographics of mechanically ventilated preterm infants prior to dexamethasone (*n* = 14).

Sex	
Male, *n* (%)	10 (71.4%)
Female, *n* (%)	4 (28.6%)
Race	
White, *n* (%)	3 (21.4%)
Black, *n* (%)	9 (64.3%)
Not specified, *n* (%)	2 (14.3%)
Birth Weight, g (SD)	772 (208)
Weight at Treatment, g (SD)	1157 (452)
Birth Gestational Age (range)	25 6/7 weeks (23 1/7–27 3/7 weeks)
Treatment Postmenstrual Age (range)	29 0.5/7 weeks (24 6/7–37 6/7 weeks)
1st sample to dexamethasone interval (d), (SD)	0.7 (1.1)
Dexamethasone initiation to 2nd sample interval (d), (SD)	2.8 (0.58)
Respiratory Severity Score (RSS)	
Pre-treatment RSS (SD)	7.21 (3.94)
Post-treatment RSS (SD)	5.28 (3.47) *
RSS reduction (SD)	1.94 (1.74)

RSS (mean airway pressure x FiO_2_), calculated on day 0 (prior to dexamethasone initiation) and on day 3 of dexamethasone course, was significantly reduced following three days of the dexamethasone treatment (* *p* = 0.0005, by two-tailed, paired Wilcoxon matched-pairs signed rank test). Data are expressed as mean (SD) or median (range) in the case of continuous variables, or number (%) in the case of dichotomous variables.

## Data Availability

The data that support the findings of this study are available from the corresponding author upon reasonable request.
